# Safety and efficacy of remimazolam in critical illness

**DOI:** 10.1186/s40560-026-00857-8

**Published:** 2026-01-23

**Authors:** Shinju Obara, Keisuke Yoshida, Satoki Inoue

**Affiliations:** Department of Anesthesiology, Fukushima Medical University, 1 Hikarigaoka, Fukushima, Fukushima 960-1295 Japan

**Keywords:** Remimazolam, Intensive care unit, Critical illness, Sedation, Mechanical ventilation, Postoperative delirium, Hemodynamic stability, Pharmacokinetics, Ventilator weaning, Procedural sedation

## Abstract

Sedation is essential in intensive care units (ICUs) for invasive procedures and mechanical ventilation; however, commonly used agents are limited by hemodynamic instability, delayed recovery of consciousness, and delirium. Remimazolam, an ultra-short-acting benzodiazepine, introduced in 2020, is rapid metabolized by hepatic carboxylesterase 1 and enables predictable recovery after prolonged administration, suggesting potential advantages for sedation in critical illness despite limited ICU-specific evidence. A narrative review was conducted based on evidence derived from randomized controlled trials, meta-analyses, and observational studies, which indicated that remimazolam provides sedation efficacy comparable to that of conventional hypnotics across surgical anesthesia, procedural sedation, and ICU settings. Multiple meta-analyses have suggested that remimazolam is associated with favorable hemodynamic tolerance and does not increase the incidence of postoperative or ICU delirium compared with propofol or other sedative agents. Delirium risk appears to be more strongly influenced by patient severity, surgical characteristics, and sedation depth than by hypnotic choice alone, although the heterogeneity across studies may partly reflect differences in delirium diagnostic tools. In ICU patients requiring mechanical ventilation, remimazolam demonstrated safety and efficacy comparable to those of propofol or midazolam, with acceptable hemodynamic stability and no consistent signs of increased mortality. Several studies have also suggested that the predictable recovery profile associated with flumazenil may facilitate ventilator weaning or recovery of consciousness, although the interpretation of recovery outcomes requires caution, particularly in studies involving routine flumazenil administration. Pharmacokinetic data in ICU populations remain limited but suggest preserved dose linearity with reduced clearance in patients with severe hepatic dysfunction. Remimazolam may be a promising sedative option for critically ill patients, offering a predictable recovery and generally favorable hemodynamic profiles. However, its optimal role in ICU sedation requires confirmation through high-quality international multicenter studies, particularly regarding prolonged mechanical ventilation, neurocognitive outcomes, and cost-effectiveness.

## Background

In the management of critically ill patients in intensive care units (ICUs), the administration of sedative agents during invasive medical interventions, such as endoscopic procedures and mechanical ventilation, is essential for alleviating patient stress. However, sedative agents have several disadvantages that are generally more pronounced in critically ill patients. For example, propofol, a representative intravenous anesthetic and sedative agent, allows precise titration of the anesthetic depth; however, its cardiovascular depressant effects frequently result in hypotension, particularly in critically ill patients, necessitating the use of vasopressors [1]. Dexmedetomidine, a selective α₂-adrenergic agonist, provides cooperative sedation; however, it may cause bradycardia and hypotension [2, 3]. Midazolam, a benzodiazepine acting on type A gamma-aminobutyric acid (GABA-A) receptors, is characterized by relative cardiovascular stability; however, it tends to accumulate in patients with hepatic dysfunction and may cause prolonged sedation in the presence of renal impairment. In addition, the accumulation of midazolam has been associated with the development of delirium [4]. Notably, benzodiazepine exposure has been consistently identified as a risk factor for delirium; therefore, the Pain, Agitation/Sedation, Delirium, Immobility, and Sleep (PADIS) guidelines [5] emphasize the importance of minimizing benzodiazepine use and implementing structured delirium management in critically ill patients.

Remimazolam is an ultra-short-acting benzodiazepine that was introduced into clinical practice in 2020. It is used for the induction and maintenance of general anesthesia as well as for procedural sedation, although the approved indications vary by country [6]. Remimazolam exerts hypnotic effects via the GABA-A receptor and is rapidly hydrolyzed by hepatic carboxylesterase 1 into a pharmacologically inactive metabolite (CNS-7054). Even after prolonged administration, remimazolam has a short context-sensitive half-time and allows predictable recovery. In addition, its sedative effects can be antagonized by flumazenil to facilitate recovery from sedation when clinically indicated. Early pharmacokinetic studies demonstrated that no clinically meaningful alterations were observed even in the presence of hepatic or renal dysfunction [7]. These properties may contribute to the reduced risk of delayed recovery of consciousness in patients with sepsis, multiorgan failure, or those undergoing dialysis. Table 1 summarizes the pharmacological characteristics of remimazolam in comparison with those of the aforementioned representative sedative agents for critically ill patients.

**Table 1:** Comparison of commonly used sedatives in critically ill patients

Agent	Primary mechanism	Respiratory effects	Hemodynamic effects	EEG and sedation profile	Advantages in the ICU	Limitations and cautions
Remimazolam	GABA_A receptor potentiation (benzodiazepine)	Dose-dependent respiratory depression	Generally mild cardiovascular depression	Beta activity with EEG slowing, characteristic of benzodiazepines; possible dissociation from BIS values	Rapid titratability; reversible with flumazenil	Limited ICU evidence; prolonged effect in severe hepatic dysfunction
Propofol	GABA_A receptor potentiation	Marked respiratory depression	Vasodilation and negative inotropy	EEG slowing to burst suppression; increased suppression ratio	Reliable and rapid deep sedation	Propofol infusion syndrome, hypertriglyceridemia, hemodynamic depression
Midazolam	GABA_A receptor potentiation (benzodiazepine)	Respiratory depression	Usually modest, but hypotension may occur	Enhanced beta activity with EEG slowing	Useful in hemodynamically unstable patients	Accumulation of active metabolites; delayed awakening
Dexmedetomidine	α2-adrenergic receptor agonist	Minimal respiratory depression	Bradycardia and hypotension	Sleep-like EEG pattern; BIS may remain low despite arousability	Suitable for non-intubated patients and weaning phase	Inadequate for deep sedation; bradycardia

*GABA_A* gamma-aminobutyric acid type A, *EEG* Electroencephalogram, *BIS* bispectral index, *ICU* intensive care unit

Collectively, the rapid onset and offset of action, along with the hemodynamic stability demonstrated in multiple studies, suggest that remimazolam has the potential to compensate for the limitations associated with conventional sedative agents. Nevertheless, evidence specific to critically ill patients, particularly in the ICU setting, remains limited, and most available data are derived from studies on procedural sedation or general anesthesia. Therefore, this narrative review aimed to examine the pharmacological background of remimazolam-based sedation in critically ill patients, with a particular focus on hemodynamic and respiratory stability and its potential impact on postoperative delirium, by drawing on currently available evidence that may be extrapolated to ICU populations.

## Literature search

A literature search was conducted on PubMed, Scopus, and the Cochrane Library to identify articles on remimazolam use in critically ill patients published between January 1, 2007, and October 10, 2025. The search terms used were “remimazolam” OR “CNS 7056” OR “ONO 2745” OR “ONO-2745” OR “ONO2745” OR "methyl 3-(8-bromo-1-methyl-6-(2-pyridinyl)-4H-imidazo(1,2-a)(1,4)benzodiazepin-4-yl)propanoate" AND “Critical Illnesses” OR “Critical Illness” OR “Critically Ill” OR "Severe Illness" OR "ICU" OR "Intensive Care." A total of 75 potential articles were found in PubMed, 140 in Scopus, and 0 in the Cochrane Library. One author (S.O.) screened the retrieved articles written in English based on the title, abstract, and full text to determine their eligibility for inclusion in this review. The studies selected for inclusion in the final narrative review are summarized in Table 2. In addition, articles selected from other sources that were considered to have important implications and studies necessary to provide a background context for this narrative review were also included. Notably, remimazolam is available in both besylate and tosilate formulations, with the latter predominantly used in China. Studies directly comparing their clinical effects are limited [8], and there is currently no evidence demonstrating clinically meaningful differences between the two formulations. Therefore, the two formulations were considered equivalent and discussed collectively in this review.

**Table 2:** Summary of key studies referenced in this review relevant to critical illness

Author (year)	Study design	Population/setting	Sample size (analyzed)	Age	ASA physical status	Intervention/exposure	Main outcome(s)	Relevance to critically ill-focused narrative
Muñoz-Carrillo et al. (2024) [9]	Systematic review and meta-analysis of randomized controlled trials	Adult patients undergoing general anesthesia for complex surgery (critically ill and non-critically ill)	9 RCTs; 1132 patients (remimazolam 678, propofol 454)			Remimazolam vs propofol for induction and maintenance of general anesthesia	Intraoperative hypotension, respiratory depression, bradycardia, injection site pain	Pooled RCT-level evidence suggesting reduced intraoperative hypotension and respiratory depression with remimazolam; however, ICU-specific and neurologic outcomes were not directly evaluated
Tong et al. (2025) [10]	Systematic review and meta-analysis of randomized controlled trials	Geriatric surgical patients undergoing general anesthesia	5 RCTs; 1,368 patients			Perioperative remimazolam vs propofol	Postoperative delirium; intraoperative hypotension; bradycardia; PONV; PACU duration	Pooled RCT evidence indicating no significant increase in postoperative delirium and a reduced incidence of intraoperative hypotension with remimazolam; ICU-specific outcomes were not directly assessed
Li et al. (2025) [11]	Systematic review and meta-analysis of randomized controlled trials	Geriatric surgical patients undergoing general anesthesia	9 RCTs; 1,132 patients (remimazolam 678, propofol 454)			Perioperative remimazolam vs propofol	Postoperative delirium; intraoperative hypotension; bradycardia; PONV; PACU duration	Summarizes pooled RCT evidence suggesting no increase in postoperative delirium and reduced intraoperative hypotension with remimazolam; ICU-specific outcomes were not directly evaluated
Zhou et al. (2025) [12]	Systematic review and meta-analysis of randomized controlled trials	Adult patients undergoing surgery under general anesthesia	17 RCTs; 3,133 patients			Remimazolam vs propofol during induction and/or maintenance of general anesthesia	Incidence of postoperative delirium	Provides pooled RCT-level evidence showing a reduced incidence of postoperative delirium with remimazolam compared with propofol; outcomes were perioperative and not specific to mechanically ventilated ICU populations
Suga et al. (2025) [13]	Systematic review and meta-analysis of randomized controlled trials	Adult patients undergoing elective surgery under general anesthesia	6 RCTs; 1,107 patients			Perioperative remimazolam vs propofol during induction and maintenance of general anesthesia	Postoperative delirium incidence; intraoperative hypotension	Pooled RCT evidence indicates that remimazolam does not increase postoperative delirium and is associated with a lower incidence of intraoperative hypotension; ICU-specific outcomes were secondary and not the primary focus
Arias et al. (2025) [14]	Systematic review and meta-analysis of randomized controlled trials	Adult patients undergoing general anesthesia or procedural sedation	23 RCTs; 3,598 patients			Remimazolam vs non-benzodiazepine hypnotics (mainly propofol)	Postoperative delirium incidence; postoperative cognitive function (MMSE)	Demonstrates no increased risk of delirium with remimazolam; ICU populations were not directly studied
Zhu et al. (2025) [15]	Retrospective cohort study with propensity score matching	Elderly patients (≥ 65 yr) undergoing elective non-cardiac surgery and admitted to the ICU postoperatively	1,652 patients (826 matched pairs after PSM)			Intraoperative remimazolam use vs no remimazolam during general anesthesia	Postoperative delirium within 7 days assessed by CAM–ICU or 3D-CAM	Large ICU-based cohort showing no association between remimazolam use and postoperative delirium
Aoki et al. (2023) [16]	Prospective cohort study	Older adults (≥ 65 yr) undergoing elective cardiovascular surgery and admitted to the ICU postoperatively	200 patients (remimazolam 78; control 122)			General anesthesia with remimazolam vs other anesthetic agents	Postoperative delirium within 5 days; delirium in ICU and during hospitalization; MMSE changes	Prospective ICU cohort showing no association between remimazolam use and postoperative delirium
Miyazaki et al. (2025) [17]	Single-center retrospective observational study	Adult patients undergoing elective off-pump coronary artery bypass surgery	36 patients (18 remimazolam; 18 sevoflurane)	72.5 (71–81) vs 73 (65–78) yr	ASA I–III (predominantly II–III)	Remimazolam vs sevoflurane for maintenance of general anesthesia	Intraoperative mean arterial pressure; norepinephrine requirement; ICU delirium (ICD–SC); ICU length of stay	Cardiac surgical ICU cohort showing more stable intraoperative hemodynamics with remimazolam without differences in ICU delirium
Harimochi et al. (2025) [18]	Prospective randomized controlled trial (open-label, single center)	Patients with severe aortic stenosis undergoing transfemoral TAVI under general anesthesia	56 patients (28 remimazolam/flumazenil; 28 sevoflurane)	86 [83–89] vs 85 [83–90] yr	ASA III–IV (predominantly III)	Remimazolam with flumazenil vs sevoflurane for maintenance of general anesthesia	Time to extubation; intraoperative hemodynamics; ICU delirium (CAM–ICU); ICU length of stay	High-risk cardiac ICU population showing faster emergence without increased ICU delirium
Kitaura et al. (2023) [20]	Single-center retrospective observational study with propensity score matching	Elderly patients undergoing transfemoral TAVR under monitored anesthesia care	253 patients screened; 152 analyzed after PSM (76 per group)	84.2 ± 5.8 vs 84.5 ± 5.1 yr (after PSM)	ASA I–IV (predominantly III–IV)	Remimazolam + remifentanil vs dexmedetomidine ± propofol + remifentanil	Time to arousal; ICU length of stay; postoperative hospital stay	High-risk TAVR population showing faster arousal with remimazolam without prolongation of ICU stay
Kim et al. (2024) [21]	Retrospective observational cohort study with propensity score matching (non-inferiority)	Patients undergoing transfemoral TAVR under monitored anesthesia care at a tertiary center	328 patients analyzed after PSM (164 remimazolam; 164 dexmedetomidine)	80.5 (76.5–84) vs 81 (77–84) yr (after PSM)		Remimazolam + remifentanil vs dexmedetomidine + remifentanil for MAC	Timely recovery defined as ICU discharge within 24 h; postoperative vasopressor/inotrope use; need for temporary pacemaker; delirium (CAM–ICU)	Severe aortic stenosis cohort showing non-inferior early ICU discharge and reduced postoperative cardiovascular support
Tang et al. (2022) [22]	Phase I open-label dose-finding study	Mechanically ventilated adult patients after non-cardiac surgery in a general ICU	36 patients	55.3 ± 13.6 yr		Continuous infusion of remimazolam besylate (0.100–0.225 mg·kg⁻^1^·h⁻^1^) with opioid analgesia	Achievement of light-to-moderate sedation (RASS − 3 to 0); rescue propofol requirement; hemodynamics; adverse events	Early ICU evidence supporting short-term postoperative sedation with stable hemodynamics
Chen et al. (2022) [23]	Prospective single-center dose–response study	Postoperative adult patients receiving invasive mechanical ventilation in a general ICU	23 patients	Median 63 (51–72) yr		Remimazolam besylate continuous infusion for ICU sedation	Achievement of light–moderate sedation (RASS − 3 to − 1); hemodynamic and respiratory stability; adverse events	Early ICU evidence supporting effective postoperative sedation under mechanical ventilation with stable hemodynamics
Tang et al. (2023) [24]	Prospective randomized controlled pilot study	Critically ill adult patients requiring deep sedation under invasive mechanical ventilation in a general ICU	60 patients (30 remimazolam; 30 propofol)	Median 63.0 (55.5–69.0) yr		Remimazolam besylate vs propofol for deep ICU sedation	Percentage of time within target deep sedation (RASS − 4 to − 5) without rescue sedation; ventilator-free hours; ICU length of stay; 28-day mortality; adverse events	Demonstrates feasibility and safety of remimazolam for short-term deep sedation in critically ill, mechanically ventilated ICU patients
Tang et al. (2022) [25]	Prospective randomized controlled pilot study	Adult patients requiring long-term invasive mechanical ventilation in a general ICU	60 patients (30 remimazolam; 30 propofol)	60.0 (51.5–66.3) vs 64.0 (55.0–69.3) yr		Remimazolam besylate vs propofol for light-to-moderate ICU sedation (RASS − 3 to 0)	Percentage of time within target sedation without rescue sedation; ventilator-free days at day 7; ICU length of stay; 28-day mortality; adverse events	Demonstrates feasibility and safety of remimazolam for long-term sedation in mechanically ventilated ICU patients
Yao et al. (2023) [26]	Single-center prospective observational study	Adult ICU patients requiring invasive mechanical ventilation ≥ 24 h	106 patients (60 remimazolam; 46 propofol or midazolam)	61.0 ± 13.5 vs 57.2 ± 16.1 yr		Continuous infusion of remimazolam vs propofol or midazolam for ICU sedation (with opioid analgesia)	ICU mortality; duration of mechanical ventilation; RASS; hemodynamics; arterial blood gas; liver and renal function; adverse events; ICU length of stay; sedation and total inpatient cost	Critically ill, mechanically ventilated cohort showing comparable ICU mortality and adverse events, with more stable physiological parameters and shorter ventilation duration under remimazolam
Turi et al. (2025) [27]	Prospective case series	Critically ill adult patients with COVID-19-related respiratory failure admitted to a tertiary ICU	5 patients	55–72 yr		Continuous infusion of remimazolam besylate for ICU sedation	Feasibility of maintaining target sedation without rescue hypnotics; hemodynamic and respiratory stability; adverse events	Preliminary ICU evidence supporting feasibility of remimazolam sedation in critically ill patients with respiratory failure
Zhao et al. (2023) [28]	Single-center retrospective observational cohort study	High-risk ICU patients with upper gastrointestinal bleeding undergoing emergency upper gastrointestinal endoscopy	88 patients (47 remimazolam; 41 propofol or midazolam)	Mean 61 ± 11.8 yr		Remimazolam-based sedation vs propofol or midazolam with opioid analgesia	Treatment-related adverse events; time to extubation; ICU length of stay; sedative cost	ICU cohort showing non-inferior safety and similar extubation time with lower sedative cost in high-risk patients
Tian et al. (2025) [29]	Single-center randomized controlled trial (single-blind, prospective)	Critically ill adult patients receiving invasive mechanical ventilation and procedural sedation in a general ICU	80 patients analyzed (40 remimazolam; 40 propofol)	65.79 ± 13.59 yr		Remimazolam besylate vs propofol for procedural sedation under mechanical ventilation	Mean arterial pressure over time; incidence of delirium (CAM–ICU); liver and renal function; blood lipid profile; ICU length of stay; sedation cost	ICU RCT demonstrating more stable hemodynamics and lower delirium incidence with remimazolam in mechanically ventilated critically ill patients
Li et al. (2025) [30]	Single-center prospective randomized controlled study	Elderly critically ill patients requiring invasive mechanical ventilation in an emergency ICU	80 patients analyzed (40 remimazolam; 40 propofol)	74.63 ± 8.10 vs 74.80 ± 9.02 yr		Remimazolam besylate vs propofol for ICU sedation under a clustered weaning strategy	In-hospital mortality; invasive mechanical ventilation time; ICU length of stay; 28-day survival; adverse events (delirium, tracheotomy)	ICU RCT in elderly, mechanically ventilated patients showing comparable safety and efficacy of remimazolam during sedation and weaning
Li et al. (2025) [31]	Systematic review and meta-analysis (PRISMA-compliant)	Elderly patients (≥ 65 yr) undergoing anesthesia or sedation, including ICU settings	8 studies; 1,641 patients			Remimazolam vs propofol	Incidence of delirium; hypotension; sleep disturbances; hypoxemia; PONV; extubation time; length of hospital stay	Shows no difference in delirium risk and improved hemodynamic stability in elderly, supporting use in high-risk and ICU-relevant populations
Liu et al. (2025) [32]	Multicenter prospective randomized controlled trial	Critically ill adult patients undergoing ventilator weaning under invasive mechanical ventilation in medical and surgical ICUs	272 patients analyzed (remimazolam 140; midazolam 132)	Mean 57.48 ± 13.99 vs 55.95 ± 15.65 yr		Sequential sedation with remimazolam besylate vs midazolam before ventilator weaning	Time to extubation; recovery time; agitation; delirium; ICU length of stay; mortality	Large ICU RCT showing faster recovery and extubation with less agitation during weaning
Grillot et al. (2025) [38]	Phase 2 single-center open-label non-randomized pilot study	Critically ill adult patients requiring ≥ 24 h of sedation in a general ICU	30 patients	Median 60 [51–70] yr		Continuous infusion of remimazolam (0.1–1 mg/min) for up to 48 h	Achievement of target sedation with hemodynamic stability during first 8 h; adverse events; pharmacokinetics	Early phase ICU study demonstrating feasibility of remimazolam sedation with acceptable hemodynamic stability
Hu et al. (2025) [39]	Prospective single-center double-blind randomized controlled PK–PD study	Adult ICU patients requiring invasive mechanical ventilation for ≥ 24 h	35 patients (PK analysis)	60.8 ± 14.0 yr		Remimazolam tosylate continuous infusion (0.1, 0.3, or 0.5 mg/kg/h) after 0.2 mg/kg loading dose	Pharmacokinetic parameters (clearance, half-life, Vd); dose–sedation relationship (RASS); hemodynamic safety; impact of liver function	Provides ICU-specific PK–PD data supporting dose selection (0.1–0.3 mg/kg/h) for mechanically ventilated patients
Chen et al. (2025) [40]	Prospective observational population pharmacokinetic study	Critically ill adult patients receiving continuous remimazolam infusion for ICU sedation (including ECMO and CRRT cases)	32 patients (236 plasma samples)	62 (26–79) yr		Continuous intravenous infusion of remimazolam besylate during ICU sedation	Population PK parameters (CL, Vd); context-sensitive decrement times; impact of ECMO, CRRT, hepatic and renal function	Provides ICU-specific PK evidence supporting predictable kinetics and suitability for long-term sedation, including during ECMO/CRRT
Eleveld et al. (2025) [41]	Translational pharmacokinetic–pharmacodynamic modeling study using pooled data	Data pooled from 20 clinical studies across anesthesia, sedation, and ICU settings	933 individuals (PK); 449 for MOAA/S PD; 613 for BIS PD	6–93 yr		Remimazolam PK–PD modeling, including covariate analysis (age, sex, opioids, hepatic/renal dysfunction, ICU, and ECMO)	Population PK–PD model; age-dependent dosing predictions; proposed target concentrations for sedation and anesthesia	Provides ICU-relevant PK–PD framework, including effects of ICU treatment and ECMO, supporting dose individualization and TCI development

Data items that could not be clearly extracted from the original publications (e.g., patient age or ASA physical status) are left blank

*ICU* intensive care unit, *RCT* randomized controlled trial, *TAVR* transcatheter aortic valve replacement, *PK–PD* pharmacokinetics–pharmacodynamics, *ECMO/CRRT* Extracorporeal Membrane Oxygenation (ECMO)/Continuous Renal Replacement Therapy (CRRT)

### Evidence derived from general anesthesia

In patients undergoing general anesthesia, including high-risk populations, the risk of hypotension associated with remimazolam and its potential benefits in patients with specific cardiovascular diseases have been increasingly evaluated. In particular, a growing body of evidence, including multiple meta-analyses, suggests that remimazolam is not associated with an increased risk of postoperative delirium. Collectively, these findings support a more favorable perspective on the use of remimazolam in critically ill patients in the ICU.

A meta-analysis of nine randomized controlled trials (RCTs) involving 1,132 adult patients undergoing complex surgery demonstrated that remimazolam was associated with significantly lower rates of intraoperative hypotension and respiratory depression than propofol [9]. This analysis provided moderate-certainty evidence for a reduced risk of hypotension (risk ratio [RR] 0.62, 95% confidence interval CI 0.50–0.76; I^2^ = 63) and respiratory depression (RR 0.28, 95% CI 0.09–0.82; I^2^ = 0). However, the RCTs included in this meta-analysis generally excluded very elderly patients and those with severe systemic disease, such as patients with an American Society of Anesthesiologists (ASA) physical status of IV or higher. With a specific focus on elderly patients, another meta-analysis including five RCTs and a total of 1,368 participants reported that remimazolam administration was associated with a significant reduction in hypotensive events compared with propofol (RR 0.55, 95% CI 0.34–0.90; P < 0.05). In contrast, no statistically significant difference was observed in the incidence of postoperative delirium (RR 0.88, 95% CI 0.58–1.33; P = 0.53) [10]. However, the certainty of the evidence was downgraded due to substantial heterogeneity across studies, particularly for hypotension, reflecting serious inconsistency in the pooled estimates. Accordingly, these findings should be interpreted with caution, especially when extrapolating to frail or critically ill populations. In a meta-analysis of 29 RCTs with 2,435 participants [11], the pooled incidence of postoperative delirium following remimazolam administration was 5% (95% CI 3–7%). The incidence of delirium was substantially higher in patients with an ASA physical status of III–IV (19%, 95% CI 15–23%) than in those classified as ASA I–II (1%, 95% CI 0–1%). Age also emerged as an important factor, and higher delirium rates were observed in children (11%, 95% CI 3–19%), followed by elderly patients (8%, 95% CI 4–13%), whereas younger adult patients exhibited the lowest incidence (1%, 95% CI 0–2%). Delirium incidence varied markedly according to the surgical type, being the highest in oncologic (16%, 95% CI 0–34%) and orthopedic surgeries (12%, 95% CI 9–14%) and the lowest in gastrointestinal and endoscopic procedures (0%, 95% CI 0–1%). Meta-regression analysis identified surgical type as the primary predictor of postoperative delirium, with orthopedic surgery associated with the highest risk compared with laparoscopic and abdominal procedures (coefficient = 0.081, P = 0.03). These findings suggest that patient severity and surgical characteristics, rather than remimazolam exposure, play dominant roles in the development of postoperative delirium. Against this background, a separate meta-analysis directly comparing remimazolam with propofol [12], which included 17 RCTs involving 3,133 patients, demonstrated that remimazolam was associated with a significantly reduced incidence of postoperative delirium (odds ratio 0.71, 95% CI 0.52–0.97; P = 0.03), with moderate heterogeneity (I^2^ = 36%). The beneficial effects were consistent across adult and older populations. According to the Grading of Recommendations Assessment, Development and Evaluation (GRADE) framework, the certainty of evidence was rated as moderate, primarily because of limitations in blinding in some of the included trials. Consistent with these findings, a meta-analysis of six RCTs (n = 1,107) showed no significant difference in postoperative delirium between remimazolam and propofol (OR 0.92, 95% CI 0.58–1.44; I^2^ = 50%), while intraoperative hypotension was significantly less frequent with remimazolam (OR 0.31, 95% CI 0.21–0.46; I^2^ = 0%). In a sensitivity analysis restricted to trials enrolling only older patients (≥ 60 years), postoperative delirium remained comparable between groups (OR 1.00, 95% CI 0.52–1.93; I^2^ = 31%) [13].

Furthermore, a comprehensive systematic review and meta-analysis that incorporated RCTs conducted under procedural sedation elucidated the relationship between remimazolam and postoperative neurocognitive outcomes. This analysis included 23 RCTs encompassing a total of 3,598 patients, of which 16 trials involved general anesthesia and seven involved sedation procedures [14]. Overall, the incidence of postoperative delirium did not differ significantly between remimazolam and non-benzodiazepine hypnotics in either general anesthesia or sedation settings (OR 1.20, 95% CI 0.76–1.91; I^2^ = 17%). Similarly, a subgroup analysis restricted to seven trials that enrolled older patients demonstrated no significant difference in delirium incidence between the treatment groups. Meta-regression analyses identified patient severity, particularly ASA physical status III–IV, as a significant determinant of delirium occurrence, whereas the choice of a hypnotic agent was not independently associated with delirium risk. Furthermore, the type of surgery significantly influenced the incidence of delirium, with major surgical procedures being associated with higher rates of delirium than intermediate and minor surgeries. Regarding postoperative cognitive function, remimazolam did not differ from the comparator hypnotics in Mini-Mental State Examination scores on postoperative days 1 and 3. However, remimazolam was associated with significantly better cognitive performance on postoperative day 7, suggesting a potential benefit for short-term postoperative cognitive recovery.

Beyond RCTs, evidence from observational studies in high-risk ICU populations has yielded highly consistent results. In a retrospective cohort study published in 2025 involving 826 propensity score-matched pairs of older patients (≥ 65 years) admitted to the ICU after non-cardiac surgery, the incidence of delirium did not differ significantly between patients receiving remimazolam and those managed with comparator anesthetics (9.3% vs. 11.3%) [15]. Similarly, the multivariable logistic regression analysis demonstrated no independent association between remimazolam use and the occurrence of delirium. In a prospective cohort study of 200 high-risk patients aged 65 years or older undergoing elective cardiovascular surgery with postoperative ICU admission, delirium assessed within 5 days after surgery using the Confusion Assessment Method for the Intensive Care Unit (CAM–ICU) and the Intensive Care Delirium Screening Checklist did not differ significantly between the remimazolam and comparator anesthetic groups (30.3% vs. 26.6%) [16].

In selected cardiac surgical populations, few studies have directly compared remimazolam with volatile anesthetics under general anesthesia, yielding findings that are largely consistent with those reported in previous meta-analyses. In a single-center retrospective study involving 36 patients with coronary artery disease who underwent off-pump coronary artery bypass surgery, patients receiving remimazolam exhibited higher intraoperative blood pressure and required lower doses of norepinephrine than those anesthetized with sevoflurane, whereas the incidence of delirium assessed using the Intensive Care Delirium Screening Checklist did not differ significantly between the groups [17]. Similarly, in a prospective randomized controlled trial of high-risk patients undergoing transcatheter aortic valve implantation, routine administration of flumazenil 0.2 mg in patients anesthetized with remimazolam significantly shortened extubation time compared with sevoflurane-based anesthesia, whereas hemodynamic parameters, respiratory outcomes, and postoperative delirium were comparable between groups [18]. Volatile anesthetics are generally associated with pronounced peripheral vasodilation and may predispose patients to hemodynamic depression, particularly during general anesthesia requiring tracheal intubation, which often requires deeper levels of hypnosis and analgesia than procedural sedation. Although volatile anesthetics have been proposed to confer myocardial protection in cardiac surgery based on experimental and clinical evidence, improvements in major clinical outcomes have not been consistently demonstrated [19]. Direct comparative evidence evaluating the cardioprotective effects of volatile anesthetics versus remimazolam remains scarce, precluding definitive conclusions regarding their relative benefits in high-risk or critically ill patients.

Overall, evidence from general anesthesia suggests that remimazolam is associated with fewer hypotensive events, while postoperative delirium rates are generally comparable to those of other hypnotics. Delirium appears to be driven more by patient and surgical factors than drug selection. Thus, evidence from general anesthesia offers useful information on the hemodynamic and neurocognitive effects of remimazolam. However, its applicability in ICU patients is limited. Surgical populations are typically managed under controlled conditions with short exposure durations and standardized anesthetic protocols, whereas critically ill patients often present with severe organ dysfunction, systemic inflammation, and hemodynamic instability. Sedation goals differ substantially; general anesthesia targets deep hypnosis, whereas ICU sedation prioritizes comfort, ventilator synchrony, and neurological assessments. In addition, the frequent use of vasopressors, opioids, and organ support therapies in the ICU may alter pharmacokinetics and pharmacodynamics. Therefore, findings from anesthesia settings should be interpreted cautiously when extrapolated to critically ill ICU populations.

### Evidence from procedural sedation

Several studies have evaluated remimazolam-based sedation in patients undergoing transcatheter aortic valve replacement (TAVR) under monitored anesthesia care (MAC), a population that typically includes older patients with severe aortic stenosis and multiple comorbidities.

In a retrospective propensity score-matched study, patients undergoing TAVR, most of whom were classified as having ASA physical status IV with an advanced New York Heart Association functional class, received either remimazolam or dexmedetomidine for MAC sedation. Remimazolam or dexmedetomidine was administered, targeting a bispectral index range of 40–70. Remifentanil 0.03 µg/kg/min was coadministered in both groups, and all procedures were performed under spontaneous ventilation. Following propensity score matching, 76 patients were included in each group. The remimazolam group required slightly higher norepinephrine doses and exhibited marginally greater respiratory depression; however, overall hemodynamic and respiratory stability were maintained. Flumazenil 0.5 mg was routinely administered at the end of the procedure, resulting in a significantly shorter time to recovery of consciousness [20]. In another retrospective observational study of high-risk TAVR patients (median age, 81 years) with propensity score matching (146 patients per group), remimazolam-based sedation demonstrated non-inferior timely recovery, defined as discharge from the ICU on postoperative day 1, compared with dexmedetomidine. Postoperative vasopressor use and temporary pacemaker implantation occurred less frequently in the remimazolam group than in the control group. The incidence of postoperative delirium did not differ significantly between the groups (remimazolam, 18.3% vs. dexmedetomidine, 18.9%) [21].

Collectively, these findings suggest that even in patients with severe aortic stenosis, who are generally considered challenging from a hemodynamic management perspective, remimazolam can achieve a depth of sedation sufficient for procedures with a certain degree of invasiveness while preserving spontaneous respiration and maintaining hemodynamic stability, thereby offering potentially useful insights into its safety profile in the management of critically ill patients. In addition, remimazolam-based MAC sedation is feasible and provides neurocognitive outcomes comparable to those of dexmedetomidine in high-risk patients undergoing TAVR. However, routine flumazenil use and heterogeneity in the study design warrant cautious interpretation.

Evidence from procedural sedation provides insights into approaches to sedation without airway protection; however, direct extrapolation to non-intubated ICU patients has important limitations. Procedural sedation is usually performed as a short, well-defined intervention in highly controlled environments with immediate airway rescue. Contrastingly, critically ill ICU patients often have limited respiratory reserves, fluctuating consciousness, and ongoing physiological instabilities. Furthermore, concomitant therapies, such as opioids, vasopressors, or noninvasive ventilation, may modify the respiratory and sedative responses. Accordingly, procedural sedation data should be viewed as supportive, but not definitive, for non-intubated ICU sedation.

### Evidence from sedation during mechanical ventilation

The efficacy and safety of remimazolam-based sedation have been investigated in the setting of mechanical ventilation in the ICU, with particular attention to hemodynamic effects, delirium, and patient outcomes. Overall, the available studies have reported outcomes that are not inferior to those observed with comparator sedatives. Several investigations have focused on identifying appropriate dosing regimens for critically ill patients.

In a phase I, prospective, open-label dose-finding study conducted in China involving mechanically ventilated patients in the ICU (n = 36; mean age 55.3 ± 13.6 years; 58.3% with comorbidities, such as hypertension), remimazolam was systematically evaluated for sedation during postoperative critical care [22]. This study demonstrated that remimazolam administered at 0.125–0.150 mg/kg/h provided rapid-onset, light-to-moderate sedation (Richmond Agitation–Sedation Scale [RASS] –3 to 0) with preserved hemodynamic stability, as reflected by a modest decrease in the mean arterial pressure (from 97 to 90 mmHg). These findings established foundational dosing evidence supporting the use of remimazolam for sedation in mechanically ventilated patients in the ICU. In a prospective dose–response study involving postoperative ICU patients receiving invasive mechanical ventilation, including older individuals (n = 23; 48% aged ≥ 65 years; 39% with chronic heart disease), remimazolam dosing that achieved light-to-moderate sedation (RASS, –3 to –1) was identified. A loading dose of 0.02–0.05 mg/kg followed by a maintenance infusion of 0.20–0.35 mg/kg/h provided satisfactory sedation while maintaining a high degree of respiratory and hemodynamic stability [23]. In an RCT involving critically ill ICU patients requiring mechanical ventilation, many of whom had acute respiratory distress syndrome (70–80%), with a median Acute Physiology And Chronic Health Evaluation (APACHE) II score of 16 and a median Sequential Organ Failure Assessment (SOFA) score of 7.0–7.5, deep sedation (RASS, –5 or –4) was administered to 30 patients in each group. Remimazolam was infused at a mean rate of approximately 0.6 mg/kg/h for a median duration of 48 h. The proportion of time during which the target sedation range was maintained without rescue sedation did not significantly differ between the remimazolam and propofol groups. In addition, no significant differences were observed in hemodynamic instability, length of ICU stay, or 28-day mortality between the two groups [24]. Overall, a dose–response relationship for remimazolam in critically ill, mechanically ventilated ICU patients may exist. However, substantial inter-individual variability has been observed, likely reflecting differences in study populations, clinical contexts, and the use of concomitant medications. Therefore, in clinical practice, careful titration based on close observation of the patient’s sedation depth and physiological status is required.

In a pilot randomized controlled trial focusing on sedation beyond 24 h during mechanical ventilation, remimazolam was compared with propofol in 60 critically ill ICU patients [25]. In this study, patients in the remimazolam group had a mean APACHE II score of 12 and a mean SOFA score of 7.2, associated with sepsis and shock in 70% and 60% of the cases, respectively. Remimazolam was administered at clinically titrated doses and provided sedation comparable to that of propofol for prolonged mechanical ventilation, with similar safety profiles and hemodynamic tolerance. No clinically meaningful differences were observed between the groups regarding sedation efficacy or major safety outcomes. In addition to randomized trials, evidence has been derived from observational studies focusing on long-term sedation during mechanical ventilation. In a prospective observational study of critically ill ICU patients requiring invasive mechanical ventilation for more than 24 h (n = 106, including 60 patients in the remimazolam group), disease severity was high, with median APACHE II scores ranging from 22 to 24. The study population included patients with intracerebral hemorrhage, shock, hepatic and renal dysfunction, and sepsis. Compared with propofol or midazolam, remimazolam was associated with smaller fluctuations in hemodynamic and respiratory parameters over time and was not associated with increased ICU mortality [26]. In a small case series of five critically ill ICU patients with COVID-19-related acute respiratory distress syndrome, remimazolam was used for sedation during mechanical ventilation and was associated with preserved hemodynamic stability throughout the infusion period [27].

During ICU management, invasive procedures may be required. A single-center retrospective cohort study evaluated 88 ICU patients who underwent upper gastrointestinal endoscopy for gastrointestinal bleeding. Most patients had multiple comorbidities and were managed by endotracheal intubation. In this setting, remimazolam-based sedation showed efficacy comparable to that of propofol or midazolam. Adverse event rates were similar between the groups, with no increase in respiratory depression, and significantly lower average sedation cost per case with remimazolam [28].

Attention has also been directed toward prolonged mechanical ventilation and delirium outcomes in ICU patients. In a single-center, randomized, prospective comparative study involving 80 critically ill patients requiring invasive mechanical ventilation and sedation (RASS –2 to 0) in the ICU (mean age 65.8 years; mean APACHE II score, 23; mean SOFA score, 7), remimazolam was associated with a significantly lower mean arterial pressure reduction than propofol. The mean arterial pressure was significantly higher in the remimazolam group on days 4 and 7 (P = 0.021 and P = 0.023, respectively), indicating superior hemodynamic stability. In addition, the incidence of delirium was significantly lower in the remimazolam group than in the propofol group (27.5% vs. 55%, P = 0.022) [29]. In contrast, another single-center randomized controlled trial enrolling 80 older patients (mean age, approximately 75 years) admitted to an emergency ICU and managed with invasive mechanical ventilation reported no significant differences in the hemodynamic parameters between remimazolam and propofol when light sedation (RASS, 0 to 1) was targeted. The duration of mechanical ventilation was comparable between the groups (propofol 108 h vs. remimazolam 105 h), and the incidence of delirium did not differ significantly (7.5% vs. 5.0%) [30]. Given the heterogeneity of patient populations and clinical settings across surgical anesthesia, procedural sedation, and ICU care, evidence integrating these contexts is particularly informative for older and high-risk patients. In this context, a recent meta-analysis including five randomized controlled trials and three retrospective studies specifically focusing on older patients (≥ 65 years) reported no significant difference in delirium incidence between remimazolam and propofol (RR 0.86, 95% CI 0.66–1.12). Notably, remimazolam was associated with superior hemodynamic stability and a lower incidence of sleep disturbance, supporting its safety profile in high-risk older adults across surgical, sedation, and ICU settings [31].

In addition to dose-finding and efficacy studies during ongoing mechanical ventilation, the effect of remimazolam on recovery and ventilator weaning has also been investigated. In a multicenter randomized controlled trial involving critically ill mechanically ventilated adults undergoing ventilator weaning, remimazolam was compared with midazolam as a sequential sedative strategy. Remimazolam was associated with a significantly faster recovery and earlier extubation, along with a lower incidence of agitation (8.6% vs. 20.5%), whereas the incidence of delirium was similar between the groups (2.9% vs. 4.6%) [32].

Overall, the available evidence indicates that remimazolam provides sedation comparable to conventional agents during mechanical ventilation in critically ill ICU patients, with acceptable hemodynamic tolerance and no signs of increased mortality. However, the effects of sedation depth and patient severity on delirium is variable, highlighting the need for high-quality trials. Although not designated as a major endpoint in the cited studies, appropriate management of spontaneous breathing during mechanical ventilation remains clinically important. For example, while the preservation of spontaneous breathing is often desirable, excessive spontaneous respiratory effort may necessitate control with agents, such as propofol [33]. Questions related to optimization of ventilatory quality would be difficult to address based on evidence derived from anesthesia settings, where fully controlled ventilation is typically employed, and therefore, further investigation in the ICU setting is required. In addition, the prolonged administration of benzodiazepine-based sedatives has been associated with the development of tolerance [34]. Emerging evidence has suggested that the potential for tolerance may be related to the accumulation of CNS7054, the primary metabolite of remimazolam [35]; whether this phenomenon may become clinically relevant in the critical care setting warrants further investigation.

### Evidence from remimazolam pharmacokinetic studies in the ICU setting

The pharmacokinetics of remimazolam have been predominantly characterized in healthy volunteers and surgical patients [36, 37]. As described in the Background section, remimazolam is rapidly metabolized and is, therefore, generally considered unlikely to accumulate, resulting in a relatively rapid offset of its clinical effects. However, Masui et al. [37] demonstrated that systemic clearance was reduced in patients with ASA physical status III or higher. Although differences in context-sensitive half-time are not considered clinically meaningful, greater insights can be obtained by focusing on the 80% context-sensitive decrement time, which more directly reflects clinical recovery. The 80% context-sensitive decrement time was defined as the time required for the effect-site concentration to decrease by 80% after the discontinuation of continuous infusion. In a 55-year-old male receiving remimazolam for 210 min, the 80% context-sensitive decrement time was approximately 65 min in patients with ASA physical status I–II, whereas it was prolonged to approximately 80 min in patients with ASA physical status III–IV. These findings suggest that although remimazolam generally allows predictable recovery, illness severity may modestly prolong drug offset and should be considered when managing prolonged infusions in critically ill patients.

In principle, pharmacokinetic findings are most directly applicable to patient populations with characteristics similar to those from which the data were derived and, therefore, cannot be fully extrapolated to critically ill ICU patients. Nevertheless, recent studies have investigated the pharmacokinetic behavior of remimazolam in mechanically ventilated ICU patients.

In a phase 2, single-center, non-randomized, open-label study conducted in France, remimazolam was administered for long-term sedation in critically ill ICU patients with heterogeneous underlying conditions, including sepsis [38]. The median Simplified Acute Physiology Score II score was 38, and the overall mortality rate was 23.3%. Remimazolam was infused targeting a RASS range from 0 to –5, for the maximum duration of 48 h. Although pharmacokinetics was evaluated as a secondary outcome, the plasma concentration profiles were relatively stable during continuous infusion and were rapidly eliminated after discontinuation of dosing. In a prospective observational study designed to evaluate pharmacokinetics (n = 35), remimazolam was administered to critically ill patients with a mean APACHE II score of 15 and mean SOFA score of 6.3. Continuous infusion of remimazolam at doses of 0.1–0.3 mg/kg/h achieved light-to-moderate sedation, and pharmacokinetic analyses demonstrated preserved dose linearity in this critically ill population [39]. Importantly, an increase in Child–Pugh score was associated with reduced clearance and prolonged elimination half-life of remimazolam, indicating that caution is warranted when administering remimazolam to patients with severe hepatic dysfunction. A population pharmacokinetic study constructed a two-compartment model based primarily on plasma concentration data collected during the elimination phase after sedation; no significant influence of extracorporeal membrane oxygenation or continuous renal replacement therapy on the pharmacokinetic parameters of remimazolam was identified, although the absence of intrainfusion sampling may limit definitive interpretation [40]. Eleveld et al. [41] developed an integrated pharmacokinetic–pharmacodynamic model for remimazolam based on a broad population of ICU patients. Although this complex model may inform future dosing tools, its predictive accuracy in ICU settings may be less consistent, indicating that careful titration to clinical effects remains essential in critically ill patients.

### Practical guidance for remimazolam administration for critically ill patients.

Remimazolam is supplied in 50-mg or 20-mg vials and is typically diluted to a concentration of 1 mg/mL for intravenous administration. According to the package insert in Japan, general anesthesia is induced at 12 mg/kg/h, followed by a maintenance infusion of 1 mg/kg/h (maximum, 2 mg/kg/h) after loss of consciousness. Supplemental boluses (0.2 mg/kg) may be administered if signs of awakening are observed. For sedation during adult gastrointestinal endoscopy, remimazolam is administered as a slow intravenous injection of 3 mg over at least 15 s, with additional doses of 1 mg administered over at least 15 s at intervals of no less than 2 min.

These dosing regimens are based on patients with relatively preserved organ function and stable hemodynamics and, therefore, represent “standard” dosing assumptions. Dose reduction according to the patient’s condition is permitted and often necessary. Even during general anesthesia, adequate hypnotic effects can frequently be achieved with doses lower than those described in the package insert. As discussed in this review, although remimazolam is generally associated with less cardiovascular depression than propofol, some degree of circulatory suppression may still occur. Thereby, it is reasonable to initiate remimazolam in critically ill patients at reduced doses and titrate carefully while closely monitoring the clinical responses.

Sedation of critically ill patients encompasses a wide range of clinical scenarios, including whether the patient is already intubated at the time of sedation, whether spontaneous breathing and a natural airway are to be maintained, whether airway protection is planned with anticipated respiratory depression, and whether the goal of sedation is to facilitate invasive diagnostic/therapeutic procedures or to maintain comfort during mechanical ventilation. Rather than defining rigid dosing protocols for each scenario, a pragmatic approach is to administer the minimum dose required to achieve the necessary level of sedation and provide appropriate respiratory and circulatory support for any resulting adverse effects.

In general, an induction dose of 12 mg/kg/h leads to a rapid increase in the effect-site concentration and may result in deep sedation and apnea within a short period of time. Although these effects are predictable and often manageable, critically ill patients may exhibit enhanced sensitivity, and multiple adverse events may occur simultaneously, complicating clinical management. Therefore, unless rapid onset of deep sedation is required, dose reduction during initiation is advisable in critically ill patients to improve overall safety. Even in non-critically ill patients, reducing the induction infusion rate to approximately 2–3 mg/kg/h often provides sufficient sedation within 10 min. Under such conditions, the sedation depth and respiratory depression progress more gradually, allowing easier titration and timely administration of vasoactive or supportive therapies. Similarly, maintenance infusion rates < 1 mg/kg/h are frequently sufficient, as suggested by the studies cited in this review.

When complete amnesia is required, such as during invasive surgical procedures, processed electroencephalographic monitoring is recommended, similar to total intravenous anesthesia. Conversely, serial assessment using the RASS is generally sufficient for ICU sedation during mechanical ventilation, where lighter sedation is appropriate. A target RASS range of –2 to 0 may be considered as a practical reference.

In patients with hepatic dysfunction, pharmacokinetic alterations may ultimately necessitate lower doses; however, the extent of such changes cannot be reliably predicted before drug administration. Therefore, at the initiation of sedation, priority should be given to achieving the intended target, followed by careful dose adjustments based on observed responses.

Continuous infusion should be discontinued at the termination of sedation. In intubated patients, extubation may be considered once the expected level of recovery is achieved, including an adequate respiratory rate, tidal volume, and recovery of muscle strength. If rapid awakening is required or recovery is markedly delayed, administration of flumazenil should be considered. However, clinicians should be aware of the risk of resedation and the potential for seizures, particularly in patients receiving benzodiazepines for seizure control, and continued monitoring is warranted.

## Future directions

In critically ill patients requiring mechanical ventilation, remimazolam has demonstrated acceptable short- to intermediate-term safety and feasibility. Nevertheless, most studies have been constrained by small sample sizes, heterogeneous populations, and relatively short exposure periods. Recovery and ventilator weaning outcomes should be interpreted cautiously, as routine flumazenil administration, which carries a risk of resedation [42], may confound the assessment of intrinsic recovery profiles. Pharmacokinetic evidence in ICU populations remains limited. Although preserved dose linearity and rapid elimination have been reported, reduced clearance has been observed in patients with severe hepatic dysfunction. Further investigation into the potential pharmacokinetic and pharmacodynamic interactions with commonly used agents is warranted in critically ill ICU patients who are frequently exposed to multiple concomitant medications.

## Conclusions

Current evidence indicates that remimazolam provides sedation efficacy comparable to that of conventional agents across general anesthesia, procedural sedation, and ICU sedation during mechanical ventilation, without consistent signs of increased hemodynamic instability or mortality. The available meta-analyses and observational studies generally suggest that remimazolam does not increase the incidence of postoperative or ICU delirium compared with other hypnotics. However, delirium outcomes may be strongly influenced by patient severity, sedation depth, surgical characteristics, and the diagnostic tools used, limiting direct comparisons across studies.

Overall, remimazolam may be considered a feasible alternative sedative for select critically ill patients; however, its optimal role in ICU practice remains unclear. Large international multicenter trials are needed to clarify its comparative effectiveness, dosing during prolonged sedation, and effects on long-term neurocognitive outcomes before broader adoption can be recommended.

## Data Availability

Not applicable.

## Abbreviations

ICU: Intensive care unit
GABA-A: Type A gamma-aminobutyric acid
PADIS guidelines: Pain, agitation/sedation, delirium, immobility, and sleep guidelines
RCT: Randomized controlled trial
ASA: American society of anesthesiologists
GRADE: Grading of recommendations assessment, development and evaluation
CAM–ICU: Confusion assessment method for the intensive care unit
TAVR: Transcatheter aortic valve replacement
MAC: Monitored anesthesia care
RASS: Richmond agitation–sedation scale
APACHE: Acute physiology and chronic health evaluation
SOFA: Sequential organ failure assessment
SAPS II: Simplified acute physiology score II
